# High-fiber-diet-related metabolites improve neurodegenerative symptoms in patients with obesity with diabetes mellitus by modulating the hippocampal–hypothalamic endocrine axis

**DOI:** 10.3389/fneur.2022.1026904

**Published:** 2023-01-17

**Authors:** Ning Luo, Yuejie Guo, Lihua Peng, Fangli Deng

**Affiliations:** ^1^Department of Endocrinology, Chenzhou No. 1 People's Hospital, Chenzhou, China; ^2^Department of Geriatrics, Chenzhou No. 1 People's Hospital, Chenzhou, China; ^3^Department of Clinical Laboratory, Chenzhou No. 4 People's Hospital, Chenzhou, China; ^4^Breast Health Care Center, Chenzhou No. 1 People's Hospital, Chenzhou, China

**Keywords:** high-fiber diet, neurodegenerative diseases, obesity, neurovascular, SPEG, AD

## Abstract

**Objective:**

Through transcriptomic and metabolomic analyses, this study examined the role of high-fiber diet in obesity complicated by diabetes and neurodegenerative symptoms.

**Method:**

The expression matrix of high-fiber-diet-related metabolites, blood methylation profile associated with pre-symptomatic dementia in elderly patients with type 2 diabetes mellitus (T2DM), and high-throughput single-cell sequencing data of hippocampal samples from patients with Alzheimer's disease (AD) were retrieved from the Gene Expression Omnibus (GEO) database and through a literature search. Data were analyzed using principal component analysis (PCA) after quality control and data filtering to identify different cell clusters and candidate markers. A protein–protein interaction network was mapped using the STRING database. To further investigate the interaction among high-fiber-diet-related metabolites, methylation-related DEGs related to T2DM, and single-cell marker genes related to AD, AutoDock was used for semi-flexible molecular docking.

**Result:**

Based on GEO database data and previous studies, 24 marker genes associated with high-fiber diet, T2DM, and AD were identified. Top 10 core genes include SYNE1, ANK2, SPEG, PDZD2, KALRN, PTPRM, PTPRK, BIN1, DOCK9, and NPNT, and their functions are primarily related to autophagy. According to molecular docking analysis, acetamidobenzoic acid, the most substantially altered metabolic marker associated with a high-fiber diet, had the strongest binding affinity for SPEG.

**Conclusion:**

By targeting the SPEG protein in the hippocampus, acetamidobenzoic acid, a metabolite associated with high-fiber diet, may improve diabetic and neurodegenerative diseases in obese people.

## 1. Introduction

The incidence of obesity is increasing annually worldwide. According to the recent data published in NEJM, the incidence of obesity has been increasing at a high rate since the 1980s, with the incidence rate being 12 and 5% among adults and children, respectively. The number of individuals with obesity is highest in China (1, 2). Obesity and type 2 diabetes mellitus (T2DM) significantly increase the incidence of neurodegenerative diseases such as depression, dementia, stroke, and memory loss (3–8). Chronic systemic inflammation throughout the body is a common feature of obesity and diabetes and may be present in the central nervous system, suggesting an important relationship among obesity, diabetes, and neurodegenerative diseases (9). Therefore, examining the regulation of energy balance in obesity and identifying biomarkers are major research directions at present.

The central nervous system plays an important role in regulating energy balance in the body, energy metabolism is also linked to the health of the central nervous system (10–14). As the main appetite control center of the hypothalamus, the arcuate nucleus region (ARC) is one of the most studied neural circuits for energy balance (10). As the sensors of peripheral nutrients and hormones, AgRP and POMC neurons in the ARC are considered key neurons involved in sensing the global energy status of an organism and playing an important role in diet and weight regulation (15–18). In a state of energy surplus, POMC neurons release neuropeptides such as α-MSH, which have appetite-suppressing effects, to reduce the energy intake of the body, thereby maintaining body weight (17). There are network loops in the hypothalamus that regulate feeding and are precisely interconnected (19). The hippocampus is part of the limbic nervous system. In addition to its relevance to cognition and learning, it has received increasing attention in the study of feeding and digestion; processing visceral sensory information and participating in the regulation of energy balance mainly through connections with the hypothalamus, amygdala and medulla (20, 21). This phenomenon may be attributed to the regulation of the hypothalamus *via* the hippocampal–hypothalamic neural loop. Direct neural projections from the ventral pole of hippocampal CA1 to hypothalamic loci are involved in the control of food intake (22). In a study, significant alterations in feeding behavior were observed in rats with a damaged hippocampus. The effects of nesfatin-1 on GD-responsive neurons in the ventral medial nucleus of the hypothalamus were significantly reduced after the hippocampal CA1 region of rats was electrically damaged, thereby affecting gastric motility (21). However, factors affecting hippocampal function are intricate. Previous studies have found that many hormone receptors are related to feeding and energy regulation in the hippocampus, such as ghrelin, nesfatin-1, and insulin (21, 23). The hypothalamic–pituitary–adrenal (HPA) axis and its neuroendocrine hormones can mediate stressful effects in the hippocampus (24). In addition, various neurovascular markers can influence hippocampal function (25). Therefore, we hypothesized that modulation of hippocampal function can improve the energy homeostasis function of the hypothalamus by improving the hippocampal–hypothalamic neural circuit, thus suppressing obesity.

An increase in dietary fiber intake is associated with a reduced risk of obesity, and dietary fiber also plays a beneficial role in obesity-related metabolic diseases (26, 27). High-fiber and low-glycaemic-index diets with conventional T2DM treatment can improve the disorder of glucolipid metabolism and have certain hypoglycaemic effects in elderly patients with T2DM (28, 29). Consumption of whole grains may prevent the development of T2DM (30, 31). Consumption of almonds increases dietary fiber intake, which is beneficial for obesity, glycaemic control and lipid profile, probably owing to the presence of fiber, which promotes an antidiabetic microbiome by increasing the amount of short-chain fatty acids (32–34). Moreover, high-fiber diet is essentially a low-calorie diet and consuming foods rich in fiber increases satiety and hence reduces caloric intake (35). In addition, a high fiber intake is associated with a reduced risk of Alzheimer's disease (AD) (36). Metabolites associated with high-fiber diet are blood markers that target key genes and suppress obesity. Inulin, a type of soluble dietary fiber, promotes the production of glucagon-like peptide-1 (GLP-1) in enteroendocrine cells and suppresses postprandial blood glucose elevation and appetite through short-chain fatty acids (SCFAs) produced by the intestinal microbiota (37, 38). Kimura et al. reported that SCFA exerts an inhibitory effect on fat accumulation *via* GPR43 (39). Therefore, high-fiber diet improves metabolic status and prevents obesity. However, its effects on the hippocampal–hypothalamic functional axis remain unknown. Previous studies have reported that dietary fiber plays a role in improving insulin resistance (40). Adherence to high-fiber diet can decrease the plasma levels of ghrelin and GLP-1 (41). Many hormone receptors related to feeding and energy regulation are present in the hippocampus, such as ghrelin, nesfatin-1 and insulin (21, 23). Therefore, we speculate that metabolites from high-fiber diet might act on the hippocampus. In order to identify new treatment targets for obesity, it is important to investigate how high-fiber diet work in obesity and the corresponding complications.

This study examined high-fiber diet' ability to delay the progression of obesity complicated by diabetes type 2 and neurodegenerative symptoms using integrated scRNA transcriptomic and metabolomic analyses, providing new insights into obesity management.

## 2. Materials and methods

### 2.1. Methylation genes in the peripheral blood of patients with T2DM–AD

Gene methylation signature matrix data associated with pre-dementia in elderly T2DM patients were downloaded from the GEO database. The following search strategy was used: keywords, “Type 2 diabetes” and “Alzheimer's disease;” study subjects, “*Homo sapiens*;” study type, “Blood methylomic signatures.” Datasets were obtained using whole-genome RNA-expression microarrays, and human-derived whole blood was used for experiments. After fine data screening, the microarray dataset GSE62003 was eventually selected (Illumina HumanMethylation 450 BeadChip; HumanMethylation 450_15017482) (42). The platform used for testing samples was GPL13534, and the dataset contained the expression data of methylation-related genes from 58 patients with T2DM. Firstly, samples with >10% missing methylation sites were excluded. Then, the R package “ChAMP” was used to perform a series of processes: the missing values were filled in using the ChAMP (43, 44). Data were extracted and screened for DEGs using the R package. The probe IDs were converted to standard gene symbols. Genes with *P* < 0.05 and |log_2_FC| > 0.1 were selected as methylation genes in peripheral blood of T2DM–AD patients.

### 2.2. Acquisition of metabolomics data for high-fiber diets

Metabolomic data of the high-fiber diet were obtained using the GEO database and a literature search, and metabolite expression matrices were created to screen and identify potential metabolic markers of serum endogenous origin based on differential expression multiplicity (|log_2_FC| > 1.00) and *t*-test (*P* < 0.05) results. The MetaboAnalyst 5.0 (http://www.metaboanalyst.ca/) database was further used to perform metabolic pathway analysis of the potential metabolic markers that were significantly back-regulated after the high-fiber diet intervention and to obtain metabolism related differentially expressed genes (DEGs) (45).

### 2.3. Downloading single-cell RNA sequencing data of hippocampal tissue from the AD patients

The high-throughput scRNA-seq data of the hippocampal samples associated with AD were downloaded from the GEO database with the following screening criteria: (i) Alzheimer's disease; (ii) human; (iii) hippocampus; and (iv) single cell RNA-seq/scRNA-seq. The single-cell sequencing data of hippocampal samples from patients with AD were extracted from the GEO database using “Alzheimer's disease” as the search term, and the scRNA-seq dataset (GSE163577) was selected for further analysis (46). The single-cell sequencing data of nine patients with AD (AD group) and eight healthy individuals (control group) in the dataset were selected, and cells with gene counts of 200–10,000 and mitochondrial gene proportion of < 5% were screened using the Seurat package as previous researches (47–52). Subsequently, the data were normalized using the “Normalizedata” function of the Seurat package and the global scaling normalization method “LogNormalize.” To remove the batch effects of cells include in the analysis and maximize the preservation of the gene expression data of these cells, the “ScaleData” function of the Seurat package was used to regress the variances of “nCount/nFeature_RNA” and “percent. Mt” (Supplementary Figure 2C). Subsequently, the “RunPCA” function of the Seurat package was used for dimensionality reduction and t-SNE clustering. The identified cells were subjected to top-down clustering analysis and annotated according to the known human gastric tissue cell marker genes.

### 2.4. Single cell sequencing quality control and data removal

For quality assessment, quality control processing of sequencing raw expression data, Limma, Seurat, Dplyr, and Magrittr packages were used. Seurat's R package is used to generate objects, remove poor quality data, and calculate the percentages of gene counts, cell counts, and mitochondrial sequencing counts from the matrix data. For quality control, set Seurat's screening criteria as follows: cells with < 3 genes expressed and < 50 genes were rejected. It was necessary to remove cells with more than 5% mitochondrial genes.

### 2.5. Cell annotation and screening of marker genes

In order to obtain the highest principal component in the cell population, further principal component analysis (PCA) was conducted after quality control of the sequencing data. The principal components were determined by *P* < 0.05 screening and the t-SNE clustering algorithm was used to select the significant components. We used the Seurat package for the t-SNE analysis, and all the removed data was classified by setting the clustering parameter of the FindClusters function in Seurat to 0.5. The R package by limma was used to adjust *P* < 0.05, and the expression change was greater than or equal to twice the (|log_2_FC| ≥ 1.00) as the criteria to filter the marker genes. In addition, candidate marker genes were found in different clusters of cells using the ggplot2 package.

### 2.6. GO/KEGG enrichment analysis of marker genes

The Gene Ontology (GO) and Kyoto Encyclopedia of Genes and Genomes (KEGG) signaling pathway analysis of the marker genes obtained in the previous step was performed in the R language to further explore the potential mechanisms of marker genes in specific cell clusters in Alzheimer's disease (53). Potential marker genes are categorized by Cellular Components (CC), Molecular Functions (MF), and Biological Processes (BP). Pathview is also used to map the corresponding signaling pathways. GO enrichment and KEGG pathway analyses were performed using the DAVID (http://david.ncifcrf.gov) and Metascape databases, and the results were visualized using the R software (54–58). The DAVID online database was conducted on methylation genes associated with high-fiber diet, T2DM, and AD for GO and KEGG enrichment analysis. Based on the *P*-value of each item *p* < 0.05, the best biological process and enrichment pathway were selected.

### 2.7. Network involving high-fiber diet, T2DM, and AD in single cells

To illustrate the relevant target sets of the T2DM-AD-monocyte marker gene methylation network, the Venn R package was used to map hippocampus-associated marker genes in AD to DEGs in high-fiber diets and differentially methylated genes in T2DM patients, respectively. In order to map intersecting protein interaction networks and output protein-interaction relationship data, the STRING database (https://string-db.org/) was applied (59–63). By analyzing the topological structure of the protein-protein interaction network model with Cytohubba plugin of Cytoscape 3.7.2, the top-ranked core targets were selected, and their degree, closeness, and betweenness values were visualized (64, 65).

### 2.8. Molecular docking

The study used AutoDock 4 for semi-flexible molecular docking of ligands and receptors to investigate the interaction of differentially metabolized compounds of a high fiber diet with the T2DM-AD-monocyte marker methylation gene (66, 67). Small molecule ligands are flexible and changeable in semi-flexible docking, while receptors are robust and difficult to change. By downloading the 3D SDF file from PubChem, modifying the structure with Chem Bio3D Ultra 14.0, and saving it as mol2 format, the active ingredient was first processed. Our next step was to download the 3D protein model of the core target from the PDB database, dehydrate, hydrogenate, extract the ligand, and save it as a PDB file using PyMOL. The files were then converted to pdbqt files using Auto Dock Tools-1.5.6. The docking results were visualized in PyMOL to map the “protein-molecule” docking interaction patterns. In addition, run Discovery Studio 2019 to find the docking site and calculate the LibDockScore. Analyze the chemical bonds formed between the docking model and the 2-dimensional image.

## 3. Results

### 3.1. Differential expression analysis of metabolites associated with high-fiber diet

**Figure 1:**
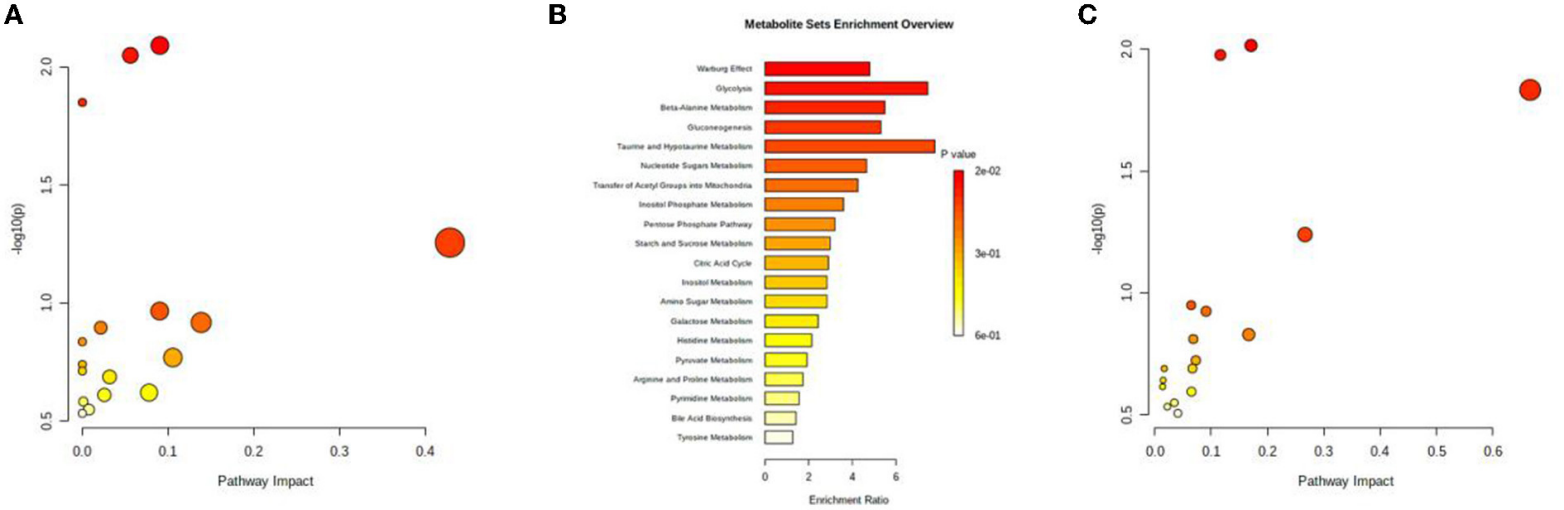
Metabolomic analysis. **(A)** Signaling pathways associated with differentially expressed metabolites associated with high-fiber diet. **(B)** Bubble map of differentially expressed metabolites associated with high-fiber diet. **(C)** Bubble map of the interaction between differentially expressed metabolites associated with high-fiber diet and DEGs.

Potential serum endogenous metabolic markers were identified *via* differential expression analysis (|log_2_FC| > 1) and a *t*-test (*P* < 0.05) using data from the GEO database and previous studies (68, 69). A total of nine upregulated and seven downregulated metabolites were identified. According to the MetaboAnalyst 5.0 database, the three most relevant pathways (*P* < 0.05) were related to the citrate cycle (TCA cycle); beta-alanine metabolism and the biosynthesis of neomycin, kanamycin, and gentamicin (Figure 1A). In addition, the enrichment analysis of potential metabolic markers revealed that their functions were mainly related to the Warburg effect, glycolysis, and beta-alanine metabolism (Figure 1B). Furthermore, the combined multifunctional analysis of high-fiber-diet-related metabolic DEGs and differential metabolites revealed that these metabolites were mainly involved in the citrate cycle (TCA cycle), beta-alanine metabolism and the biosynthesis of neomycin, kanamycin, and gentamicin, which is consistent with the results of the previous pathway enrichment analysis (Figure 1C). As a result of the above analysis, we identified differential metabolites associated with high fiber diets.

### 3.2. Methylation genes in the peripheral blood of patients with T2DM–AD

The expression profiles of methylation-related genes in the peripheral blood of patients with T2DM–AD were obtained. And a total of 11 upregulated and 10 downregulated methylation-related genes were identified and visualized on a heat map (Supplementary Figure 1A). The transcriptomic data of these DEGs were imported into the R software to construct a volcano plot (Supplementary Figure 1B). The peripheral blood of T2DM-AD patients was analyzed for methylation-related genes.

### 3.3. Cellular distribution and characteristics in the hippocampal tissue of patients with AD

**Figure 2:**
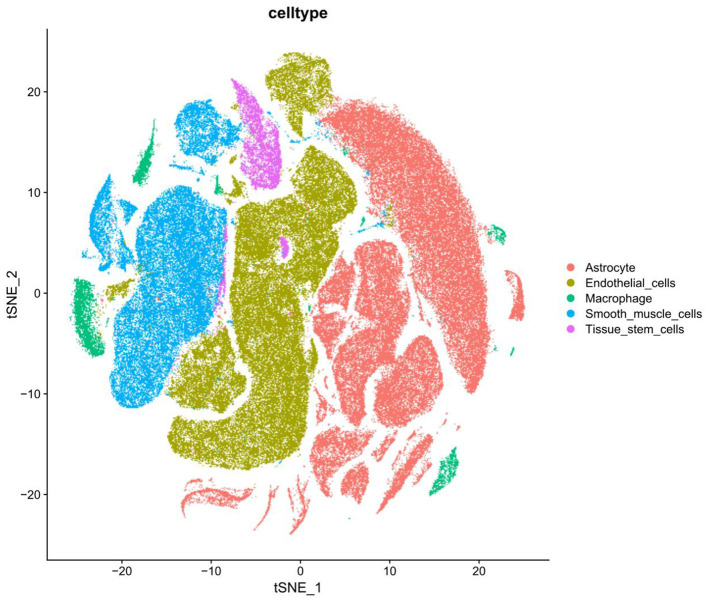
Annotated distribution maps of cells in tSNE map.

Hippocampal samples from patients with AD were extracted from the GEO database using “Alzheimer's disease” as the search term, and the scRNA-seq dataset (GSE163577) was selected for further analysis. The single-cell sequencing data of nine patients with AD and eight healthy individuals in the dataset were selected, and cells with gene counts of 200–10,000 and mitochondrial gene proportion of < 5% were screened using the Seurat package in R (Supplementary Figures 2A, B). To remove the batch effects of cells include in the analysis and maximize the preservation of the gene expression data of these cells, the “ScaleData” function of the Seurat package was used to regress the variances of “nCount/nFeature_RNA” and “percent. Mt” (Supplementary Figure 2C). Subsequently, the “RunPCA” function of the Seurat package was used for dimensionality reduction and t-SNE clustering (Supplementary Figures 3A, B). A total of 182,056 cells and 27,005 associated genes were identified after quality control, integration and normalization of data and removal of batch effects from the single-cell sequencing data of patients with AD and healthy individuals. The identified cells were subjected to top-down clustering analysis and annotated according to the known human gastric tissue cell marker genes. A total of 27 cell types were identified (Supplementary Figure 3B), with uniform scattered distribution and good integration of batch effects. The expression of marker genes in each cell type was specific, indicating that the cell annotation results were accurate (Figure 2). Thus, hippocampal tissue of AD patients was examined with respect to its distribution and characteristics.

### 3.4. Screening and enrichment analysis of marker genes

**Figure 3:**
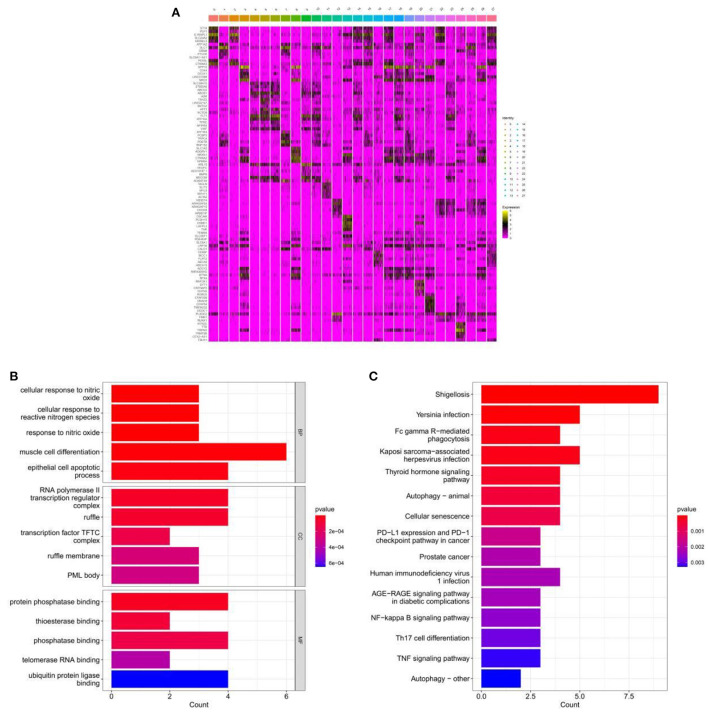
Enrichment analysis for the identification of markers genes. **(A)** Heat map demonstrating the distribution of marker genes. **(B)** Histogram of GO enrichment analysis of marker genes. **(C)** Histogram of KEGG functional analysis of marker genes.

Based on the results of cell annotation, different types of cell subpopulations were identified using the Seurat package, and DEGs in different cell subpopulations in the hippocampal tissue of patients with AD and healthy individuals were screened using the FindMarkers function. A total of 12,610 DEGs were identified and visualized on a heat map (Figure 3A). GO analysis revealed that the identified DEGs were mainly enriched in cellular responses to nitric oxide, muscle cell differentiation, and RNA polymerase II transcriptional regulation complex (Figure 3B). KEGG analysis revealed that the genes were mainly enriched in pathways associated with autophagy, cellular senescence, AGE–RAGE signaling in diabetic complications and NF–kappa B signaling (Figure 3C).

### 3.5. Interaction network of high-fiber-diet-related metabolites, methylation-related DEGs associated with T2DM–AD, and single-cell marker genes associated with AD

**Figure 4:**
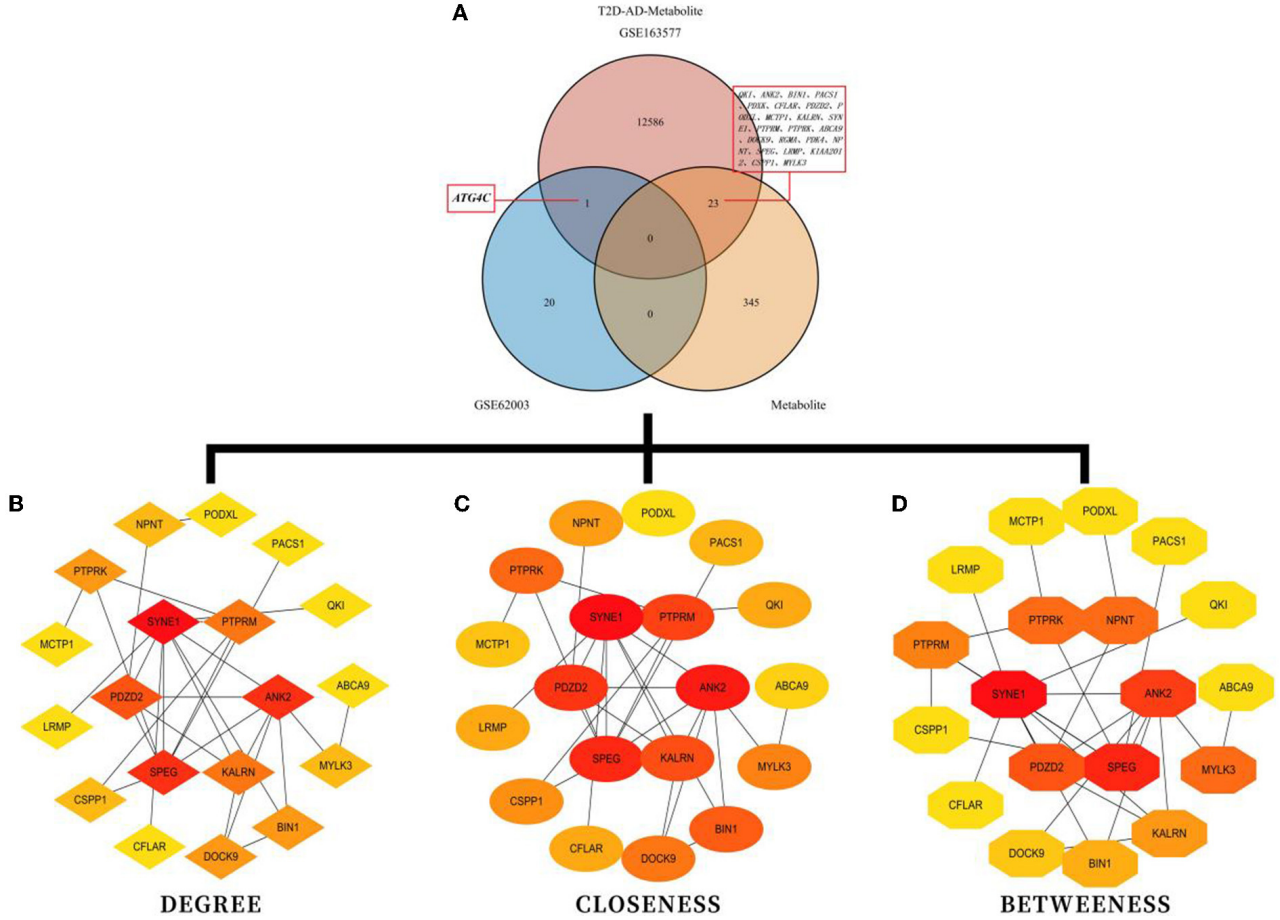
Enrichment analysis for the identification of markers genes. **(A)** Venn diagram demonstrating high-fiber-diet–T2DM–AD-related marker genes. **(B)** Potential core target genes identified based on degree values. **(C)** Potential core target genes identified based on closeness values. **(D)** Potential core target genes identified based on between-ness values.

**Figure 5:**
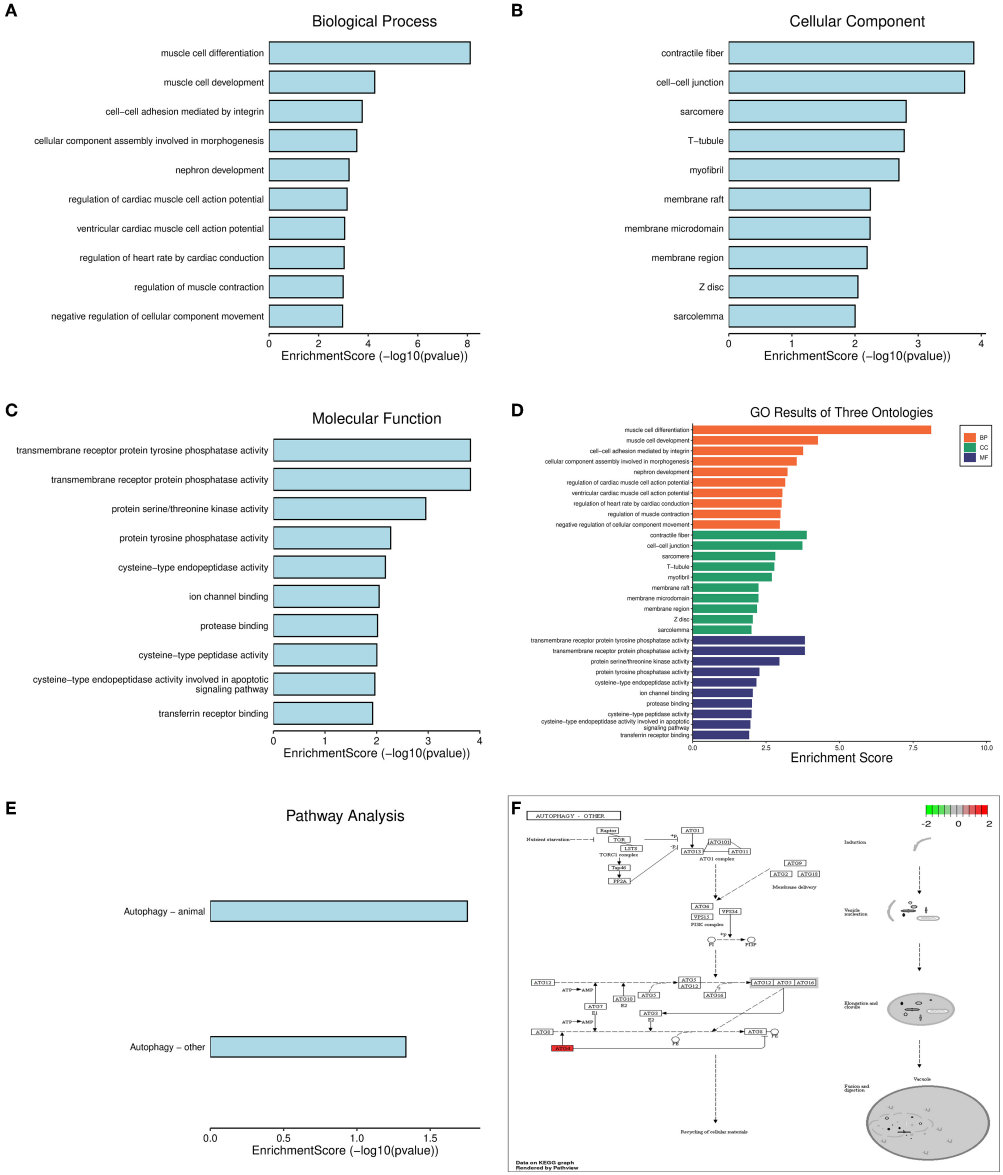
GO and KEGG enrichment analysis. **(A)** Histogram of BP functional analysis. **(B)** Histogram of CC functional analysis. **(C)** Histogram of MF functional analysis. **(D)** Histogram of GO enrichment analysis. **(E)** Histogram of KEGG enrichment analysis. **(F)** Autophagy signaling pathway.

There were 24 high-fiber-diet–T2DM–AD marker genes identified by intersecting high-fiber-diet-related metabolites with methylation-related DEGs associated with T2DM (Figure 4A). The STRING (version 11.0) database was used to construct a gene interaction network for these marker genes. The species selected was “Homo sapiens.” To identify hub genes (Figures 4B–D), the cytoHubba plug-in in Cytoscape 3.7.2 software was used to construct a protein–protein interaction network based on network topology, calculate degree, closeness, and betweenness values, and select the size of the values for ranking. Top hub genes included SYNE1, ANK2, SPEG, PDZD2, KALRN, PTPRM, PTPRK, BIN1, DOCK9, and NPNT. As a result, we constructed a network of interactions between high-fiber diet-associated metabolites, methylation-associated DEGs associated with T2DM-AD, and single-cell marker genes related to AD. Functional and pathway enrichment analyses of the 24 high-fiber-diet–T2DM–AD-related marker genes were performed using Metascape. GO enrichment analysis revealed a total of 245 biological processes (BPs), 33 cellular components (CCs), and 26 molecular functions (MFs). BPs included muscle cell differentiation, muscle cell development, cell–cell adhesion mediated by integrin, CC assembly involved in morphogenesis, and nephron development. MFs included transmembrane receptor protein tyrosine phosphatase activity, transmembrane receptor protein phosphatase activity, protein serine/threonine kinase activity, protein tyrosine phosphatase activity, and cysteine-type endopeptidase activity. CCs included contractile fibers, cell–cell junction, sarcomere, T-tubules, and myofibrils (Figures 5A–D). According to KEGG pathway enrichment analysis, the marker genes were enriched in two pathways (Figures 5E, F), of which autophagy was the pathway of interest in this study.

### 3.6. Molecular docking validation

**Figure 6:**
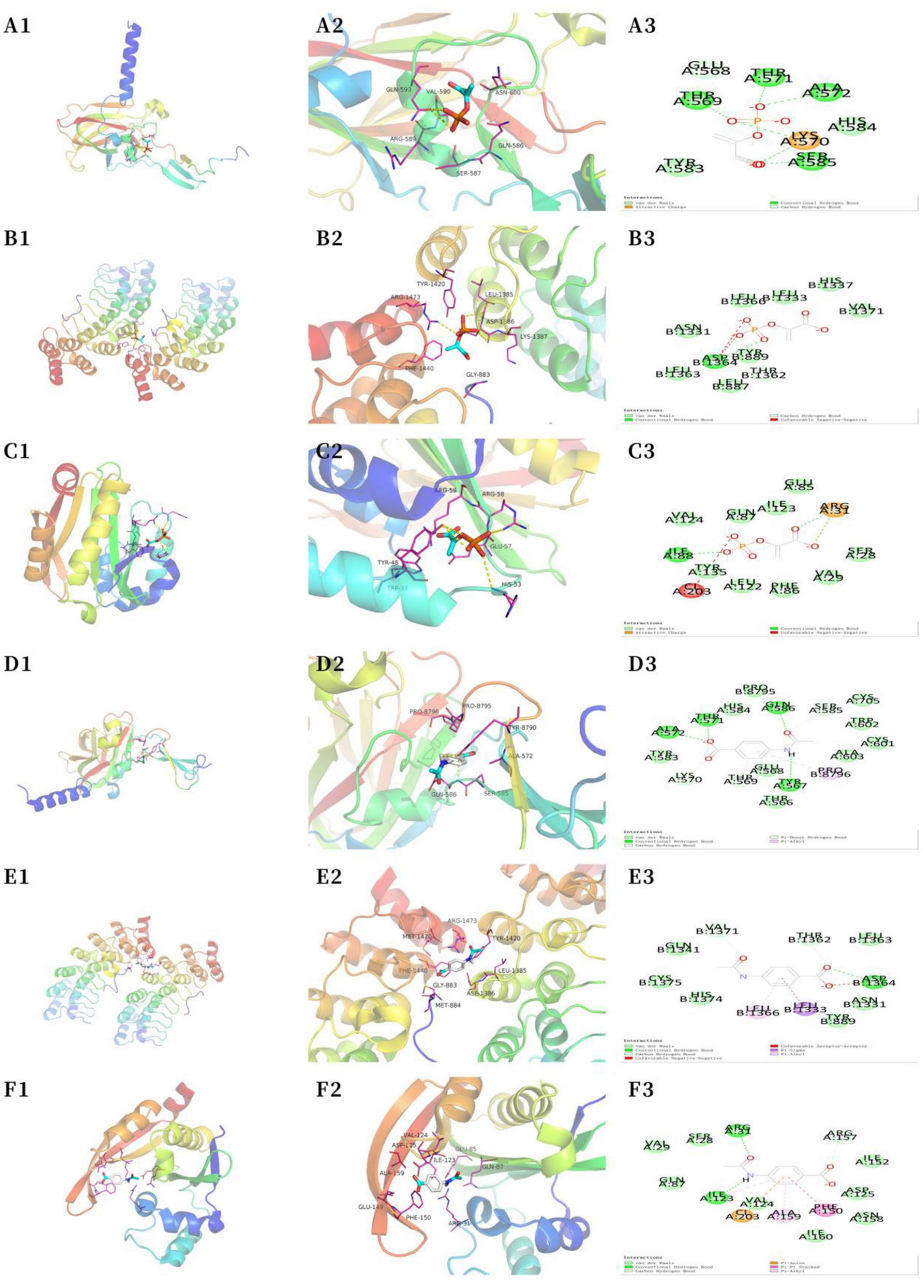
Molecular docking analysis. **(A)** SYNE1 phosphoenolpyruvate (macroscopic) **(A1)**; SYNE1 phosphoenolpyruvate (microscopic) **(A2)**; SYNE1 phosphoenolpyruvate **(A3)**. **(B)** ANK2 phosphoenolpyruvate (macroscopic) **(B1)**; ANK2 phosphoenolpyruvate (microscopic) **(B2)**; ANK2 phosphoenolpyruvate **(B3)**. **(C)** SPEG phosphoenolpyruvate (macroscopic) **(C1)**; SPEG phosphoenolpyruvate (microscopic) **(C2)**; SPEG phosphoenolpyruvate **(C3)**. **(D)** SYNE1 acetamidobenzoic acid (macroscopic) **(D1)**; SYNE1 acetamidobenzoic acid (microscopic) **(D2)**; SYNE1–acetamidobenzoic acid **(D3)**. **(E)** ANK2–acetamidobenzoic acid (macroscopic) **(E1)**; ANK2–acetamidobenzoic acid (microscopic) **(E2)**; ANK2–acetamidobenzoic acid **(E3)**. **(F)** SPEG–acetamidobenzoic acid (macroscopic) **(F1)**; SPEG–acetamidobenzoic acid (microscopic) **(F2)**; SPEG–acetamidobenzoic acid **(F3)**.

The most significantly altered metabolic markers, including phosphoenolpyruvate and acetamidobenzoic acid, as well as the top three core proteins, SYNE1 (4DXR), ANK2 (5Y4E), and SPEG (6CY6) (the corresponding structural domains of the proteins are mentioned in parentheses) were analyzed with the AutoDock Tools (version 1.5.6) software. Smaller the binding energy, the stronger the binding capacity. Table 1 shows that phosphoenolpyruvate and acetamidobenzoic acid had adequate binding affinities for the core proteins SYNE1 (4DXR), ANK2 (5Y4E), and SPEG (6CY6). Acetamidobenzoic acid had the strongest binding affinity for the core protein SPEG (6CY6), with an RMSD value of < 2.00 (Figure 6). Docking the active molecule with the corresponding target protein in Discovery Studio 2019 revealed that acetamidobenzoic acid binds *via* hydrogen and hydrophobic bonds to SPEG (6CY6). Acetamidobenzoic acid forms hydrogen bonds with amino acid residues at position 31 (ARG) and 123 (ILE) and hydrophobic bonds with amino acid residues at position 159 (ALA) and 150 (PHE) of the structural domain of SPEG (6CY6) (Figure 6). Acetamidobenzoic acid, a metabolite associated with high-fiber diet, may target SPEG in the hippocampus and affect autophagy related pathway.

**Table 1:** Molecular docking and binding energy.

**Protein**	**Compound**	**Structure**	**DS (LibDockScore)**	**Vina (kcal·mol^−1^)**	**RMSD**
SYNE1 (4DXR)	Phosphoenolpyruvate	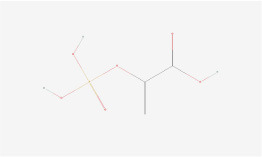	67.3832	−2.9	1.276
ANK2 (5Y4E)	Phosphoenolpyruvate	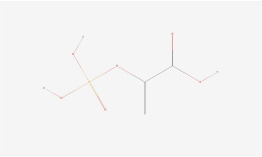	44.551	−3.5	1.491
SPEG (6CY6)	Phosphoenolpyruvate	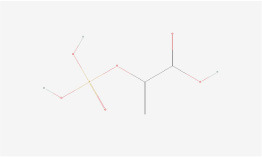	57.2527	−4.5	1.011
					
SYNE1 (4DXR)	Acetamidobenzoic acid	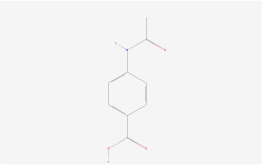	90.7005	−4.9	2.728
					
ANK2 (5Y4E)	Acetamidobenzoic acid	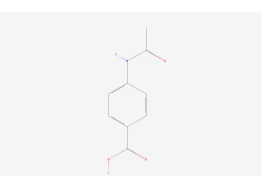	56.6351	−4.5	1.450
					
SPEG (6CY6)	Acetamidobenzoic acid	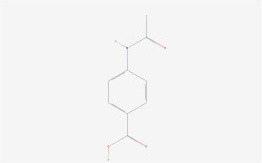	75.5652	−6.0	1.719

## Discussion

In this study, metabolites and signaling pathways associated with high-fiber diet were identified based on databases. The GEO database was used to identify 10 core high-fiber-diet–T2DM–AD-related marker genes, whose functions are mainly related to autophagy. The results of molecular docking suggested that high-fiber-diet-associated metabolites can stably bind to the core high-fiber diet–T2DM–AD-associated proteins, with the most stable binding observed between SPEG (6CY6) and acetamidobenzoic acid. These results suggest that high-fiber diet influences autophagic homeostasis in the hippocampus through binding of the metabolite acetamidobenzoic acid to the SPEG (6CY6) protein and regulation of the hippocampal–hypothalamic endocrine axis, eventually improving the diabetic and neurodegenerative disease states of patients with obesity.

Autophagy is involved in the regulation of lipid metabolism, and its dysregulation in adipose tissue is associated with the development of metabolic diseases (70, 71). Dysregulation of autophagy alters energy metabolism in hypothalamic neurons and white adipose tissue (WAT). Imbalance of autophagy in hypothalamic neurons can lead to increased caloric intake and weight gain, resulting in obesity and metabolic disorders (72). Mitochondrial autophagy is critical for protecting neurons in the hippocampal CA1 region from ischaemic stress injury (73). In addition to hippocampal CA1 neuronal deletion leading to cognitive impairment, direct neural projections from the ventral pole of hippocampal CA1 to hypothalamic loci are involved in the control of food intake (22, 74). Furthermore, the autophagic pathway is closely related to the pathogenic mechanism underlying the impairment of intestinal mucosal barrier function (75). Impairment of intestinal barrier structure and function is an important pathogenic process in T2DM (76). Autophagy is also involved in the pathological process of AD through several mechanisms, such as the removal of misfolded proteins, and is a novel therapeutic target for AD (77, 78). Therefore, autophagy is closely related to the pathogenesis of obesity and the associated metabolic diseases T2DM and neurodegenerative diseases and regulates the hippocampal–hypothalamic neuroendocrine axis. In the present study, KEGG pathway enrichment analysis strongly suggested that the function of high-fiber-diet–T2DM–AD-related DEGs was mainly related to autophagy, which is consistent with the results of previous similar studies (79–82). Therefore, metabolites associated with high-fiber diet may play a role in the pathological process of T2DM and AD among patients with obesity by affecting autophagy in the hippocampus and hypothalamus.

Furthermore, this study demonstrated that the high-fiber-diet-associated metabolite acetamidobenzoic acid can bind to the SPEG (6CY6) protein in the hippocampus and affect autophagic homeostasis in the hippocampus, thus improving the diabetic and neurodegenerative disease states of individuals with obesity. Acetamidobenzoic acid is a derivative of benzoic acid. Benzoic acid derivatives are involved in promoting the activity of the autophagy–lysosome pathway (83). Benzoic acid is involved in the composition of the compound Mn (III) tetrakis (4-benzoic acid) porphyrin chloride (MnTBAP), which reduces obesity by reducing adipocyte hypertrophy and adipogenesis and regulating energy balance and improves insulin function (84, 85). Benzoic acid derivatives present in garlic shells can be combined with other compounds to synergistically activate the PPAR signaling pathway or inactivate the phospholipase D signaling pathway to exert an anti-T2DM effect (86). In addition, the bifunctional molecule BPBA synthesized using benzoic acid can target Aβ and inhibit neuroinflammation, which plays a role in AD (87). To the best of our knowledge, this study is the first to identify the potential role of acetamidobenzoic acid in the pathogenesis of obesity–T2DM–AD, thus laying a foundation for the subsequent development of new drugs.

Striated muscle preferentially expressed protein kinase (SPEG) is a myosin light-chain kinase containing a double serine/threonine kinase domain and multiple immunoglobulin (Ig)-like and proline-rich regions involved in protein–protein interactions (88). SPEG as a single gene can be alternatively spliced into several tissue-specific isoforms, including BPEG (the brain) and SPEGα (skeletal muscle) and SPEGβ (cardiac muscle) (89–91). Patients with neurodegenerative diseases had significantly lower levels of SPEG methylation, which is strongly associated with obesity. The m6A demethylase FTO may regulate adipocyte differentiation and adipogenesis by regulating the expression of proteins such as gastric starvation hormone, pro-adipogenic factors and peroxisome proliferator-activated receptor, thereby affecting the development of obesity (92). Furthermore, FTO plays a major role in the development of T2DM, as m6A methyltransferases can inhibit adipogenesis, delay the onset and progression of obesity by inhibiting autophagosome formation, blocking mitotic clone expansion, and controlling adipogenic differentiation in mesenchymal stem cells (93). And missense mutations in SPEG are also closely associated with the development of T2DM in GK rats (94). Therefore, acetaminobenzoic acid may target SPEG in the hippocampus, thereby affecting the autophagic balance of the hippocampus and regulating the hippocampal-hypothalamic endocrine axis, improving diabetic and neurodegenerative disease states.

To the best of our knowledge, this study is the first to report that the SPEG protein in the hippocampus, a peripheral blood biomarker in patients with obesity with concomitant T2DM and neurodegenerative diseases, can bind to acetamidobenzoic acid, a high-fiber-diet-related metabolite, and plays an important role in the development of T2DM and neurodegenerative diseases in patients with obesity. In addition to acetamidobenzoic acid and SPEG, the metabolite phosphoenolpyruvate, which is significantly altered by high-fiber diet, was found to have a strong binding affinity for the core proteins SYNE1 and ANK2, suggesting that high-fiber diet can benefit individuals with obesity through multiple targets and pathways.

However, this study has some limitations. This study was mainly based on bioinformatic analysis of data extracted from databases and lacks relevant experimental validation. Further research is needed to determine exactly how acetamidobenzoic acid binds to hippocampal SPEG proteins.

## 5. Conclusion

Acetamidobenzoic acid, a metabolite associated with high-fiber diet, can target SPEG (6CY6) protein in the hippocampus, thereby affecting autophagic homeostasis in the hippocampus, regulating the hippocampal–hypothalamic endocrine axis and eventually improving the diabetic and neurodegenerative disease states of patients with obesity.

## Data availability statement

The original contributions presented in the study are included in the article/Supplementary material, further inquiries can be directed to the corresponding author.

## Ethics statement

Ethical review and approval was not required for the study on human participants in accordance with the local legislation and institutional requirements. Written informed consent from the patients/participants or patients/participants' legal guardian/next of kin was not required to participate in this study in accordance with the national legislation and the institutional requirements.

## Author contributions

LP: conceptualization, methodology, software, investigation, formal analysis, and writing—original draft. YG: data curation, writing—original draft, methodology, software, investigation, and formal analysis. FD: visualization and investigation. NL: conceptualization, funding acquisition, resources, supervision, and writing—review and editing. All authors contributed to the article and approved the submitted version.

## Supplementary material

Supplementary Figure 1 **(A)** Heat map of differentially expressed methylation-related genes in the GSE62003 dataset. **(B)** Heat map of differentially expressed methylation-related genes in the GSE62003 dataset (the red color represents upregulated gene expression, and the green color represents downregulated gene expression).

Supplementary Figure 2 **(A)** Quality control and data removal plots for the GSE163577 dataset. **(B)** Distribution of the top 10 most significant genes in the GSE163577 dataset. **(C)** Distribution of genes before and after normalization of data in the GSE163577 dataset.

Supplementary Figure 3 **(A)** Principal component analysis distribution map. **(B)** t-SNE principal component distribution map.
