# Supplementary figures and images for: High-fiber-diet-related metabolites improve neurodegenerative symptoms in patients with obesity with diabetes mellitus by modulating the hippocampal–hypothalamic endocrine axis
Source: Front Neurol. 2023 Jan 17;13:1026904. doi: 10.3389/fneur.2022.1026904 (PMC9888315; doi:10.3389/fneur.2022.1026904)

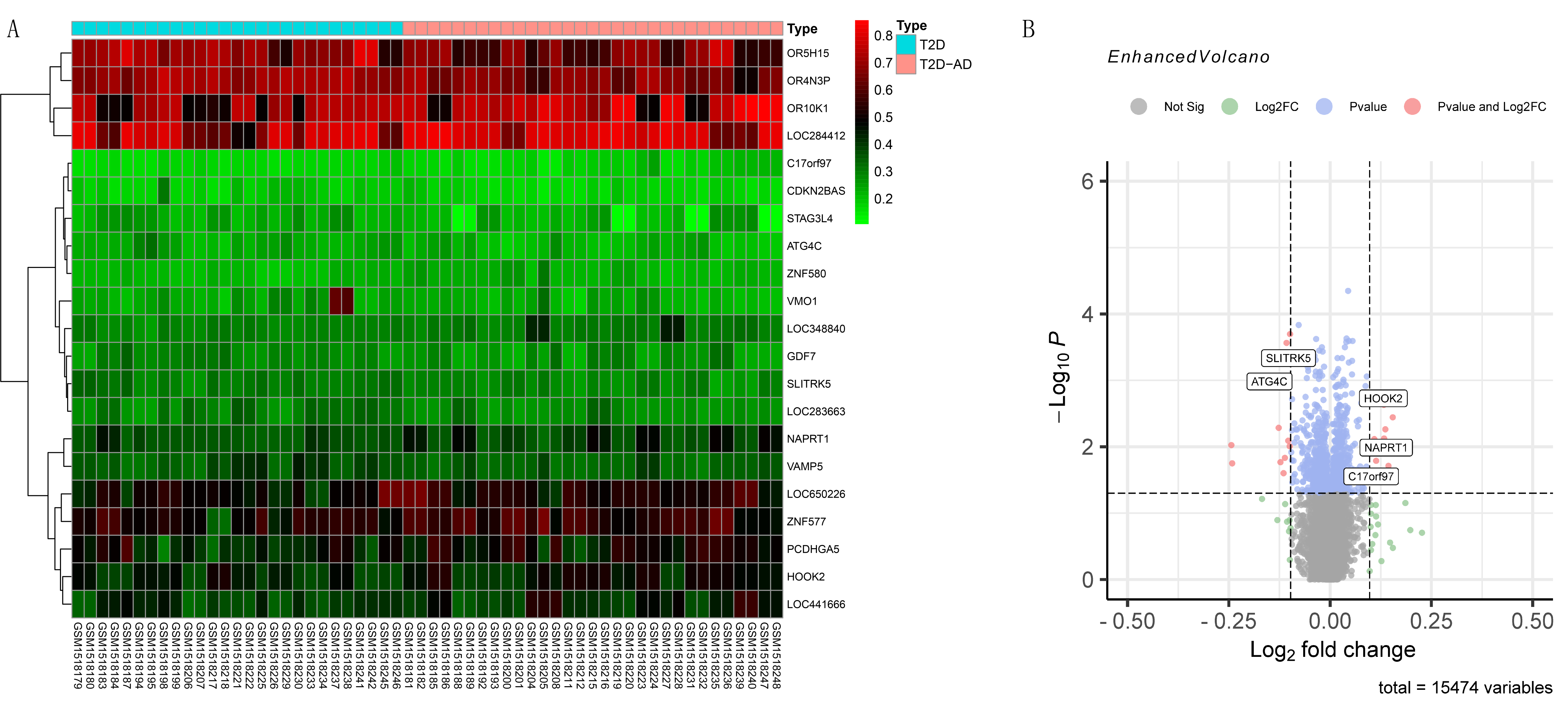

Supplement: Supplementary Figure 1 — (A) Heat map of differentially expressed methylation-related genes in the GSE62003 dataset. (B) Heat map of differentially expressed methylation-related genes in the GSE62003 dataset (the red color represents upregulated gene expression, and the green color represents downregulated gene expression). [file Image_1.TIF]

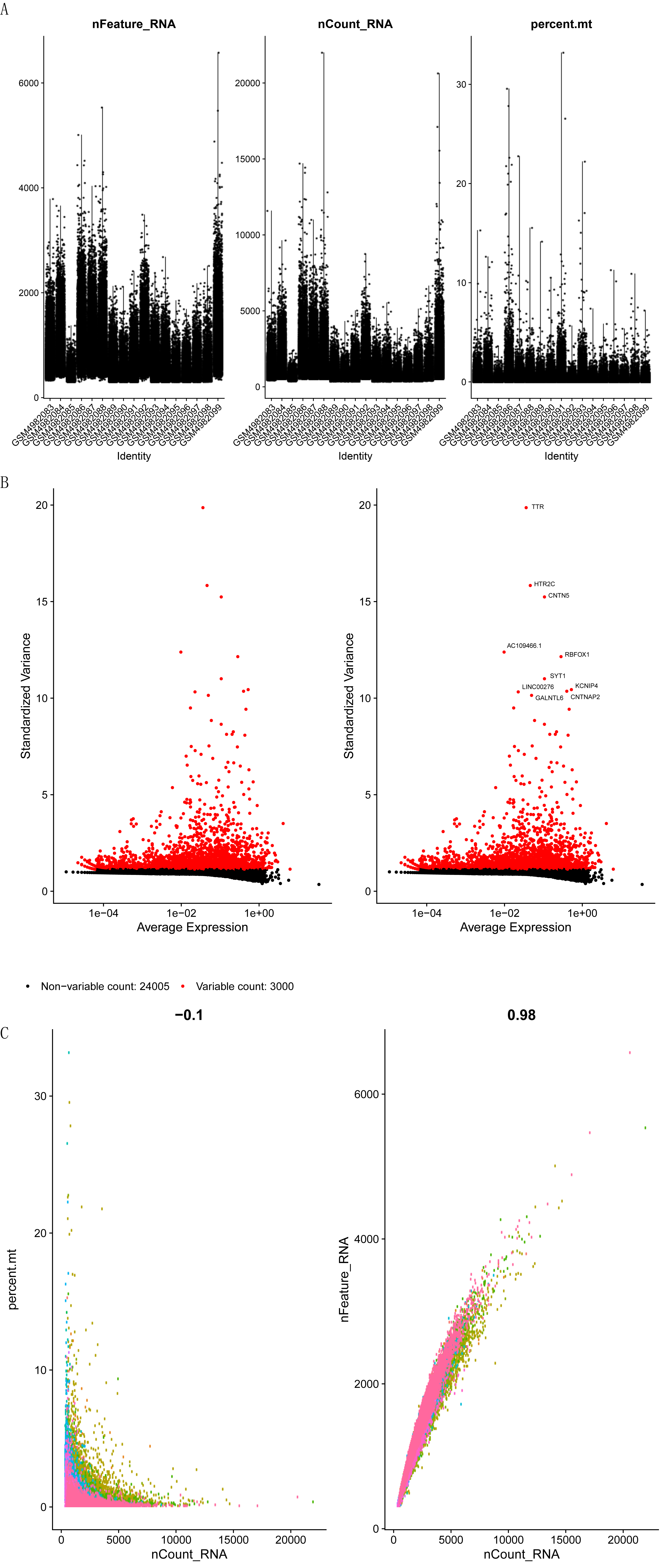

Supplement: Supplementary Figure 2 — (A) Quality control and data removal plots for the GSE163577 dataset. (B) Distribution of the top 10 most significant genes in the GSE163577 dataset. (C) Distribution of genes before and after normalization of data in the GSE163577 dataset. [file Image_2.TIF]
